# Variation of foliar silicon concentrations in temperate forbs: effects of soil silicon, phylogeny and habitat

**DOI:** 10.1007/s00442-021-04978-9

**Published:** 2021-07-14

**Authors:** Marius Klotz, Jörg Schaller, Susanne Kurze, Bettina M. J. Engelbrecht

**Affiliations:** 1 Department of Plant Ecology, Bayreuth Center of Ecology and Environmental Research (BayCEER), University of Bayreuth, 95440 Bayreuth, Germany; 2 Leibniz Centre for Agricultural Landscape Research (ZALF), 15374 Müncheberg, Germany; 3 Smithsonian Tropical Research Institute, Apartado 0843-03092, Balboa, Ancon, Republic of Panama

**Keywords:** Temperate grassland, Dicotyledonous plant species, Phylogenetic signal, Drought resistance, Phytoliths

## Abstract

Silicon (Si) accumulation is known to alleviate various biotic and abiotic stressors in plants with potential ecological consequences. However, for dicotyledonous plants our understanding of Si variation remains limited. We conducted a comparative experimental study to investigate (1) interspecific variation of foliar Si concentrations across 37 dicotyledonous forbs of temperate grasslands, (2) intraspecific variation in foliar Si concentration in response to soil Si availability, the influence of (3) phylogenetic relatedness, and (4) habitat association to moisture. Foliar Si differed markedly (approx. 70-fold) across the investigated forbs, with some species exhibiting Si accumulation similar to grasses. Foliar Si increased with soil Si availability, but the response varied across species: species with higher Si accumulation capacity showed a stronger response, indicating that they did not actively upregulate Si uptake under low soil Si availability. Foliar Si showed a pronounced phylogenetic signal, i.e., closely related species exhibited more similar foliar Si concentrations than distantly related species. Significant differences in foliar Si concentration within closely related species pairs nevertheless support that active Si uptake and associated high Si concentrations has evolved multiple times in forbs. Foliar Si was not higher in species associated with drier habitats, implying that in dicotyledonous forbs of temperate grasslands high foliar Si is not an adaptive trait to withstand drought. Our results demonstrated considerable inter- and intraspecific variation in foliar Si concentration in temperate forbs. This variation should have pervasive, but so far understudied, ecological consequences for community composition and functioning of temperate grasslands under land-use and climate change.

**Supplementary Information:**

The online version contains supplementary material available at 10.1007/s00442-021-04978-9.

## Introduction

Silicon (Si) accumulation has been shown to be beneficial to plants by alleviating abiotic stressors such as drought, heavy metals and salt (Cooke and Leishman 2016), deterring mammalian and insect herbivores (Massey et al. 2007), and reducing the risk of pathogen infections (Fauteux et al. 2005). Ecological research on Si has mostly focussed on grasses, because Si concentrations are considered to be lower in dicotyledonous than monocotyledonous angiosperms (Katz 2014). However, dicots also exhibit substantial variation in plant Si concentration (Hodson et al. 2005; Ishizawa et al. 2019; Deshmukh et al. 2020). Indeed, dicots cover a comparable range of foliar and shoot Si concentrations as grasses (0–11% Si per dry weight; Hodson et al. 2005), suggesting that Si uptake and accumulation mechanisms vary across dicots with possibly pervasive ecological consequences (Katz 2014, 2019; Deshmukh et al. 2020). However, environmental conditions vary substantially within and across available studies (and often are not specified), and most studies include only a few species and/or small sample sizes. In addition, unaccounted variation due to sampling of different plant organs (shoot, leaves or whole plant), variation in plant age and analytical methods likely contribute to the observed variation of Si concentrations in dicots. Therefore, the extent to which the observed variation of Si concentrations in dicots is due to species-specific differences or to environmental factors currently remains elusive. Here, we conducted a comparative experimental study to investigate (1) interspecific variation of foliar Si across dicotyledonous forb species of temperate grassland habitats, (2) their intraspecific response to soil Si availability, the role of (3) phylogenetic relatedness and (4) habitat association to moisture.

Plant Si concentration can be affected by the availability of Si as silicic acid (H_4_SiO_4_) in the soil, which originates from weathering of primary minerals as well as dissolution of secondary clay minerals and biogenic compounds (Schaller et al. 2021). Multiple studies that experimentally manipulated soil Si as well as field studies on soils with different soil Si availability demonstrated that higher available soil Si can lead to higher plant Si concentrations (Mitani and Ma 2005; Liang et al. 2006; Melzer et al. 2010; Quigley et al. 2017). The size of the effect differs among species (Schaller et al. 2018; Ishizawa et al. 2019), which likely reflects differences in their Si uptake mechanisms. Plant roots take up Si passively by diffusion, actively by ATP-consuming transporters, or by employing a combination of both mechanisms, leading to variation in Si uptake (Mitani and Ma 2005; Liang et al. 2006; Ma and Yamaji 2015; Deshmukh and Bélanger 2016). Silicon is then transferred with the transpiration stream through the xylem into the stems and leaves, where it accumulates and polymerizes to amorphous silicon dioxide (Epstein 1999). Species that passively take up Si accumulate only low amounts of Si in leaves (Mitani and Ma 2005). The regulation of passive Si uptake is only possible indirectly (e.g., through changes in transpiration or carbon allocation) and is, therefore, limited. Species with predominantly passive Si uptake should, ﻿therefore,﻿ exhibit an increase of foliar Si in response to increased soil Si availability. In contrast, ATP dependent active uptake leads to high Si accumulation and allows plants to directly regulate Si uptake (Ma and Yamaji 2015). Even when soil Si availability is low, species with active Si uptake should be able to reach high foliar Si concentrations and gain potential associated ecological benefits (Faisal et al. 2012). Their response to higher soil Si availability should, therefore, be less pronounced. We thus expect that across multiple species, the response in foliar Si concentrations to soil Si availability should be negatively correlated with the species’ capacity to accumulate Si.

Silicon transporter proteins and associated genes have been identified throughout the plant kingdom (Trembath-Reichert et al. 2015; Ma and Yamaji 2015; Deshmukh and Bélanger 2016). Uptake mechanisms are heritable (Strömberg et al. 2016), and plant Si concentrations exhibit a pronounced phylogenetic signal, i.e., closely related taxa show more similar Si concentrations than distantly related taxa (Hodson et al. 2005; Trembath-Reichert et al. 2015; Katz 2015; Strömberg et al. 2016). At high taxonomic levels, non-vascular plants reach higher Si concentrations than vascular plants, angiosperms have higher Si concentrations than gymnosperms and within angiosperms, most monocot orders have higher Si concentrations than dicot orders (Hodson et al. 2005; Trembath-Reichert et al. 2015). Silicon concentrations also differ across orders within dicots (Hodson et al. 2005; Katz 2015). However, meta-analyses of species-level phylogenetic signals within dicots have been largely precluded by the limited comparability of available studies (see above). Furthermore, species selections were not targeted to address evolutionary and ecological hypotheses considering the species’ phylogenetic relatedness. To address this gap, we tested whether variation of foliar Si concentrations across dicotyledonous forb species raised under standardized conditions correlates with their phylogenetic relatedness and/or with their ecological habitat association to moisture.

High Si accumulation has been suggested to be adaptive in dry habitats (Quigley and Anderson 2014; Strömberg et al. 2016; Katz 2019), because Si has been shown to alleviate drought stress in plants (reviewed in Cooke and Leishman 2016 and Chen et al. 2018). Several mechanisms may underlie these effects; Si can enhance water uptake via an increased root:shoot ratio, stronger osmotic driving force and/or enhanced aquaporin activity, which may allow plants to maintain higher transpiration rates under drought stress (Hattori et al. 2008; Chen et al. 2018). Silicon can also indirectly alleviate drought stress by reducing oxidative damage (Gong et al. 2005). However, the ecological and evolutionary significance of foliar Si for plant drought resistance remains elusive, especially in dicots. If Si accumulation is indeed adaptive in dry habitats, we expect that across phylogenetically constrained species pairs (i.e., species pairs within genera or families), forb species associated with drier habitats should consistently exhibit higher foliar Si concentrations than species associated with moister habitats.

Dicotyledonous forbs can constitute a major part of plant diversity in grasslands, which cover about 43% of the earth’s surface, harbour an outstanding biodiversity and offer valuable ecosystem services (Gibson 2009). Species-specific habitat association to moisture is an important driver for community assembly and diversity in grasslands (Silvertown et al. 2015). For predicting consequences of increasing intensity and frequency of droughts, i.e., periods of low water availability, on grasslands under climate change (IPCC 2013), it is important to improve our understanding of Si variation in forbs and the associated phylogenetic and ecological drivers.

We conducted a comparative greenhouse experiment with congeneric and confamiliar species pairs of temperate eudicotyledonous forbs (referred to as dicots), with each pair including a species associated to high and low habitat moisture. All species were cultivated on Si-enriched soil to compare species’ maximum Si accumulation capacity, and a subset of species was additionally grown under low Si availability to investigate intraspecific responses to soil Si. We addressed the following hypotheses: (1) dicot forb species growing under standardized environmental conditions vary substantially in their foliar Si concentrations, reflecting differences in Si uptake mechanisms; (2) higher soil Si availability increases foliar Si concentrations, with the species’ responses being negatively related to their capacity to accumulate Si; (3) plant Si concentrations exhibit a phylogenetic signal, i.e., closely related species show more similar foliar Si concentrations than distantly related species; and (4) foliar Si concentrations are higher in species associated with dry than with moist habitats.

## Materials and methods

### Study species and phylogeny

We focused on 37 species from 24 genera, 18 families and 14 orders, including 13 congeneric and three confamiliar species pairs, and five additional species (Table S1). Species were selected based on the following criteria: (1) species pairs included contrasting moisture associations based on Ellenberg’s indicator values [moisture (M)-value; Ellenberg et al. 2001] with M-values differing by at least two levels within pairs, and M-values for species from drier and moister habitats ranging from 3 to 5 and 5 to 8, respectively; (2) maximizing phylogenetic diversity, i.e., choosing species from different taxonomic orders; (3) species associated to open habitats (i.e., we excluded species of shaded habitats); and (4) commercial availability of seeds (Rieger-Hofmann GmbH, Blaufelden, Germany). Seed production aimed to maintain the genetic diversity of the species' source populations in southern Germany.

We generated a phylogenetic tree of the study species based on the highly resolved species-level megaphylogeny by Zanne et al. (2014). For six study species, which were not included in Zanne et al. (2014), we used phylogenetic data of congeneric species as replacement (for details see Table S1). We generated the phylogeny using the R-package “ape” (Paradis and Schliep 2019).

### Experiment

Five individuals per species and soil Si treatment were included (see Table S1 for a few exceptions). Seeds were germinated in multicell plug trays with low Si soil in a greenhouse (Bayreuth, Germany). Seedlings were individually transplanted into separate pots (Deepot Cells, Stuewe & Sons, Oregon, USA; diameter: 6.5 cm, depth: 36 cm) with the experimental substrates (see below). Seedlings of all species were transplanted within 1 day at cotyledon stage or after development of first foliage leaves.

The experimental substrates consisted of 60% sieved sandy topsoil, 30% quartz sand and 10% clay granulate (hereafter referred to as soil). For high Si availability the soil was enriched with 12.5 g of amorphous Si (Aerosil 300, Evonik Industries AG, Essen, Germany) per litre substrate and thoroughly homogenized. Addition of Aerosil 300 does not lead to changes of soil pH (Schaller, unpublished data). In the low Si treatment, no Si was added. The resulting plant-available soil Si concentration was 110 mg kg^−1^ for the high Si soil and 40 mg kg^−1^ for the low Si soil (for analysis see below), equivalent to high and intermediate levels of plant-available Si in agricultural soils (Caubet et al. 2020).

We watered all plants regularly by hand and fertilized them four times during the experiment (0.3% NPK fluid fertilizer, Wuxal Super, Wilhelm Haug GmbH & Co. KG, Ammerbuch, Germany). Temperatures ranged between 18 and 22 °C and plants grew under natural light intensity supplemented with artificial greenhouse light (Plantstar 400 W E40, Osram, Munich, Germany). The position of species and treatments in the greenhouse was randomized and rearranged regularly.

We harvested the aboveground biomass of the plants 10 weeks after transplantation in a randomized order. Leaves of each individual were cleaned, removing any residual soil material, oven-dried for 48 h at 65 °C, and ground for analyses.

### Foliar and soil Si analyses

Silicon was extracted from the leaves for 5 h by an alkaline method using 30 mg of leaf material and 30 ml of 0.1 M sodium carbonate solution (Na_2_CO_3_) in a regularly shaken water bath following Struyf et al. (2010). The solution was subsequently passed through a 0.2 µm syringe filter (ChromafilXtra CA-20/25). Soluble soil Si was extracted in CaCl_2_ following Schaller et al. (2018). Three g of soil were shaken with 30 ml of 0.01 M CaCl_2_ for 1 h at ambient laboratory temperature. The suspension was centrifuged (8000×*g*, for 10 min) and the supernatant decanted.

The Si concentration of the leaf or soil extract was determined with inductively coupled plasma optical-emission spectrometry (ICP-OES) using a Varian Vista-Pro Radial element analyser (Varian Inc., Palo Alto, USA), compare Schaller et al. (2018).

### Statistical analyses

#### Interspecific differences in foliar Si, and intraspecific responses to soil Si

Interspecific differences of foliar Si across all 37 species grown on the high Si soil were analysed using one-way ANOVA with a Tukey HSD post-hoc test using the R-package “agricolae” (de Mendiburu 2020). Differences of foliar Si within each of the 16 congeneric or confamiliar species pairs were analysed using one-way ANOVA. To analyse the effect of soil Si availability on foliar Si concentrations we calculated a two-way ANOVA with species (26 species), treatment (low vs. high Si) and their interaction as explanatory factors. Furthermore, we tested for differences of foliar Si concentrations within each individual species by one-way ANOVA. Foliar Si concentrations were log_10_ transformed to fulfil normality and homoscedasticity. We excluded one outlier, an individual of the high Si treatment (*Verbascum lychnitis*), which we were able to trace back to soil contamination.

To evaluate whether intraspecific responses of foliar Si to soil Si are related to their foliar Si concentration under high soil Si availability, representing the species’ maximum Si accumulation capacity, we calculated a response ratio for each species based on (untransformed) mean values of foliar Si in each treatment (RR_Foliar Si_; compare Hedges et al. 1999) as: RR_Foliar Si_ = log_10_ (foliar Si_high Si_/foliar Si_low Si_). A more positive RR_Foliar Si_ indicates a higher increase of foliar Si concentrations in response to higher soil Si availability. We tested the relationship between the species’ RR_Foliar Si_ and their foliar Si concentrations in the high Si treatment with Spearman rank correlation (*n* = 26). To assess whether species ranking of Si concentrations stays consistent under different soil Si availabilities, we additionally calculated the Spearman rank correlation coefficient (ρ) of foliar Si concentrations between the low and high Si treatment (*n* = 26).

To ease data interpretation, we classified species based on their foliar Si concentrations under high soil Si availability into low-accumulating species (< 5 mg g^−1^), assumed to predominantly take up Si by passive diffusion, and high-accumulating species (> 5 mg g^−1^), assumed to additionally take up Si actively through ATP-consuming Si transporters (compare Strömberg et al. 2016 for a similar approach; threshold values based on Ma et al. 2001).

### Phylogenetic signal in foliar Si

To analyse the phylogenetic signal in the species’ mean foliar Si concentrations in the high Si treatment (i.e., their maximum accumulation capacity), and in RR_Foliar Si_, we separately calculated Pagel’s lambda λ (Pagel 1999) for each parameter (across 32 and 26 species, respectively) using the function “phylosig” from the R-package “Phytools” (Revell 2012). A Pagel’s lambda λ = 1 indicates that phylogeny can fully explain the variation in foliar Si concentrations (or RR_Foliar Si_), i.e., a pure Brownian model of evolution, and λ = 0 indicates that phylogenetic relationships cannot explain the variation. We separately tested the null hypotheses of λ = 0 and λ = 1 using likelihood-ratio tests.

### Relationship of foliar Si to species moisture association

We tested the relationship between species’ association to habitat moisture (M-value, Ellenberg et al. 2001) with foliar Si (under high Si) across 32 species (all species pairs) and with RR_Foliar Si_ across 26 species. We calculated ordinary least square regressions (OLS), and additionally conducted phylogenetic generalized least square regressions (PGLS) using the R-packages “nlme” (Pinheiro et al. 2020) and “ape” (Paradis and Schliep 2019) to account for species phylogenetic relatedness. We used two variance–covariance structures in the PGLS models, each representing a potential evolutionary trajectory along the branches of the phylogeny; either evolution by strict Brownian motion or Brownian evolution adjusted by Pagel’s λ as scaling parameter (Pagel 1999), with the scaling parameter λ obtained using maximum likelihood. Since the models based on strict Brownian motion consistently showed the poorest fit (highest AIC values), we only included the results of λ-modified PGLS models.

We additionally tested the relationship of foliar Si to the species’ association to habitat moisture separately for the subset of species pairs containing only low-accumulating species, and for the subset of species pairs containing at least one high-accumulating species, using OLS and PGLS regression.

All statistical analyses were performed in R version 4.0.2 (R Core Team 2020).

## Results

### Interspecific differences in foliar Si, and intraspecific responses to soil Si availability

**Fig. 1:**
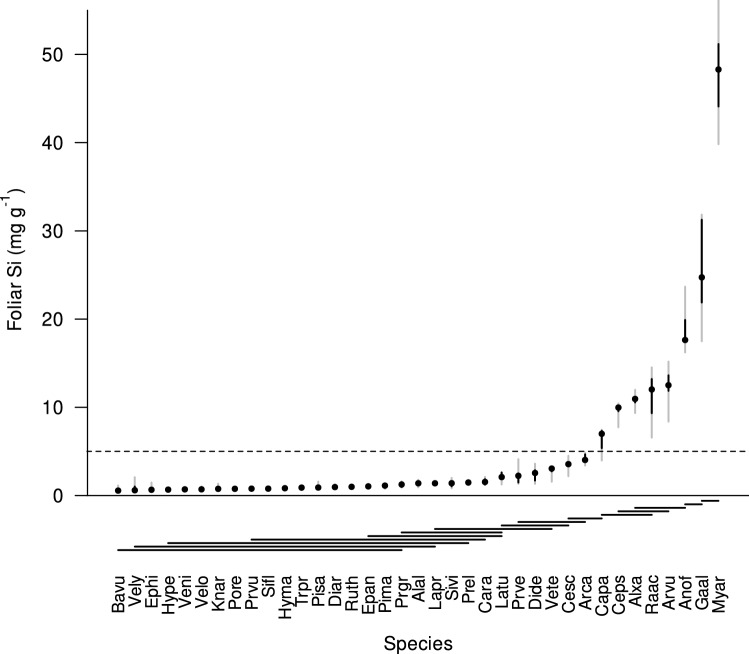
Foliar Si concentrations of 37 forb species cultivated on Si enriched soil. Species are sorted by increasing Si concentrations. The median is given as point, interquartile range as black line, and the 2.5–97.5% percentile as grey line. *N* is usually 5, see Table S1 for details. Species significantly differed in foliar Si (based on ANOVA of log_10_-transformed foliar Si concentrations; *P* < 0.001). Results of post-hoc comparison are indicated as horizontal lines with each line indicating species groups which did not significantly differ from each other. The horizontal dashed line indicates the threshold value (5 mg g^−1^) for low- vs. high-accumulating species. See Table S1 for species abbreviations

Foliar Si concentrations varied significantly across the 37 forb species (*P* < 0.001, *F*_*3*6,144_ = 91.17) growing under high Si availability (Fig. 1), with about 70-fold interspecific variation. Overall, 20% of species (8 species out of 37) showed high accumulation of Si (foliar Si > 5 mg g^−1^). The remaining species (80%, 29 species) exhibited low Si accumulation (foliar Si < 5 mg g^−1^).

**Fig. 2:**
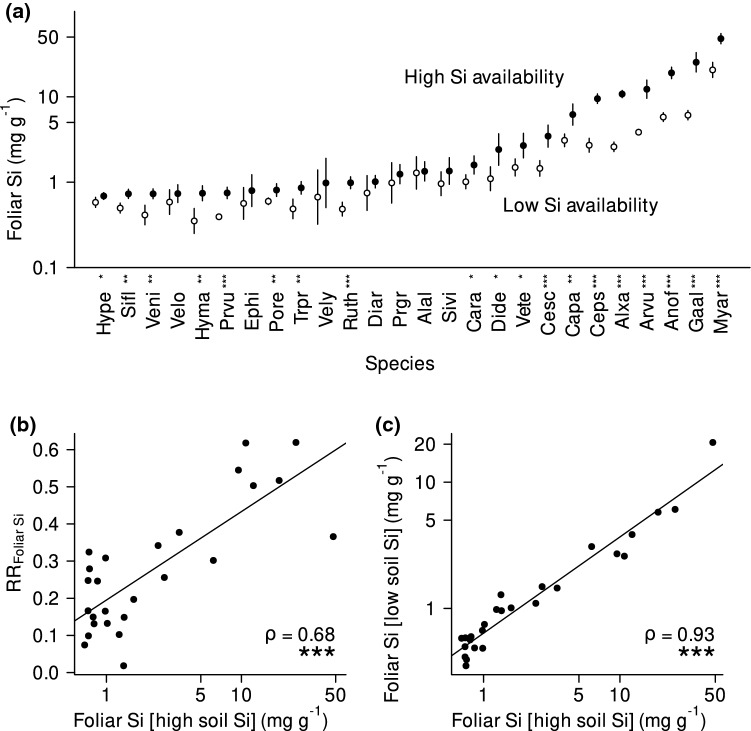
**a** Foliar Si concentrations under low (open circles) and high (solid circles) soil Si availability in 26 forb species (mean ± standard deviation). **b** Correlation between the species’ responses to increased soil Si availability (RR_Foliar Si_) and their foliar Si concentrations under high soil Si availability, reflecting their maximum Si accumulation capacity, and **c** correlation between foliar Si concentrations under high and low soil Si availability across species. In **a** asterisks indicate significant differences within species (based on ANOVAs; ***P < 0.001, **P < 0.01, *P < 0.05; *n* = 5; for details see “Results” and Table S2). In **b** and **c**, the Spearman rank correlation coefficients (ρ) are given, and asterisks indicate significance levels. The solid lines in **b** and **c** represent fitted linear regressions for visualization. Note that both axes in **c**, the *y-*axis in **a** and the *x-*axis in **b** are log_10_-scaled. See Table S1 for species abbreviations

Foliar Si increased with higher soil Si availability, with the effect size of soil Si varying across species (Fig. 2a; soil Si availability: *P* < 0.001, *F*_1,206_ = 338.91; species:* P* < 0.001, *F*_25,206_ = 163.72; soil Si availability × species: *P* < 0.001, *F*_25,206_ = 4.37). Overall, 73% of species (19 of 26 species) exhibited significantly higher foliar Si concentrations under higher soil Si availability, with up to a fourfold increase of foliar Si between low and high soil Si (Table S2).

The intraspecific response of foliar Si to soil Si availability (RR_Foliar Si_) was positively related to the species’ maximum Si accumulation capacity, i.e., species with higher Si concentrations under high Si availability responded stronger to the change in Si availability (Fig. 2b). Species ranking of foliar Si concentrations remained consistent across soil Si availability, i.e., foliar Si concentrations of the species were significantly related between low and high soil Si (Fig. 2c).

### Phylogenetic signal

**Fig. 3:**
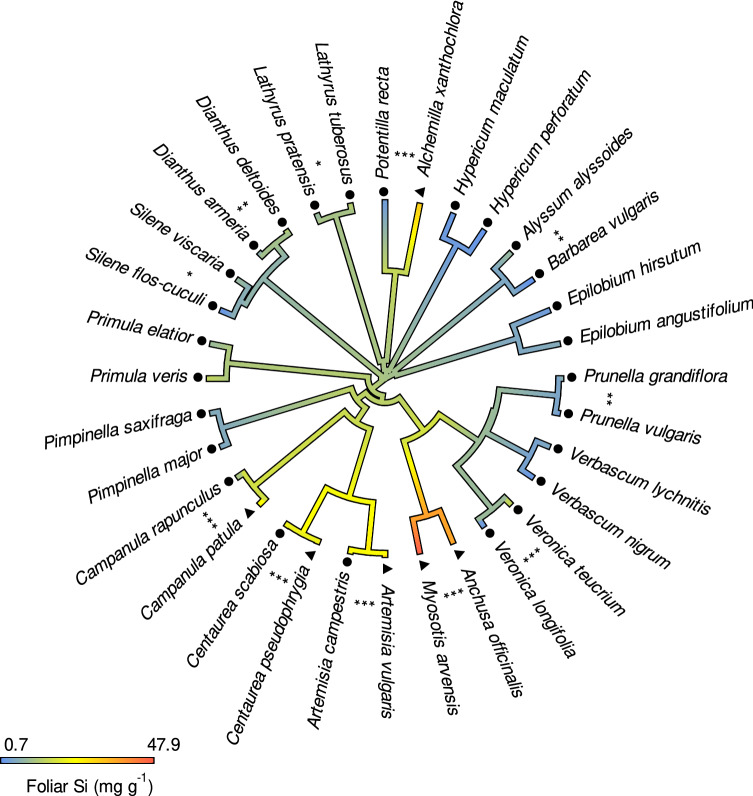
Interspecific variation in foliar Si concentrations in 32 forb species mapped on a phylogenetic tree (based on Zanne et al. 2014), with colours indicating foliar Si concentrations. Differences of foliar Si within species pairs, based on ANOVAs are indicated by asterisks (***P < 0.001, **P < 0.01, *P < 0.05; *n* = 5, see Table S3 for details). High-accumulating species are indicated by triangles, and low-accumulating species by circles

Foliar Si concentrations exhibited a strong phylogenetic signal of λ = 0.93, which differed significantly from both 0 and 1 (*P* = 0.002 and *P* < 0.001, respectively), in the plants growing on high Si soil (Fig. 3). The strong phylogenetic signal in the full set of species pairs was mainly driven by the species pair that exhibited by far the highest foliar Si concentrations (Boraginaceae, *Myosotis arvensis* and *Anchusa officinalis*). After removing this species pair, the phylogenetic signal decreased to λ = 0.39, which still differed significantly from 1 (*P* < 0.001) but not from 0 (*P* = 0.08). Consistently, the closely related species within 11 of the 16 congeneric or confamiliar pairs showed significantly different foliar Si concentrations (Fig. 3, Table S3), with four of the species pairs including a high- and a low-accumulating species. The phylogenetic signal of RR_Foliar Si_ (λ = 0.48) in the subset of species grown on low and high Si soil also differed significantly from 1 (*P* < 0.001) but not from 0 (*P* = 0.314).

### Association to habitat moisture

**Fig. 4:**
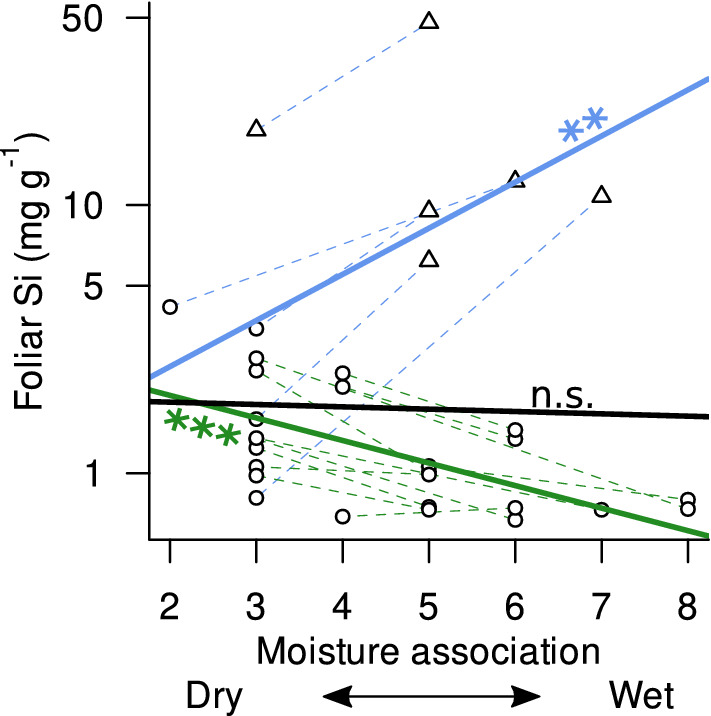
Relationship between the species’ association to habitat moisture (Ellenberg *M *value) and foliar Si concentrations across 32 species in 16 congeneric and confamiliar species pairs. The best overall model (PGLS) was not significant (*P* = 0.8, continuous black line), but a significant positive relationship emerged in species pairs including a high Si accumulator (*P* = 0.002, blue continuous line; triangles denote high Si accumulators), and a significant negative relationship in species pairs containing only low Si accumulators (*P* < 0.001, green continuous line; circles denote low Si accumulators). Dashed lines connect species within phylogenetically constrained pairs, with colours denoting the two species subsets. For details, see Table S4. Note that the *y*-axis is log_10_-scaled

Species’ foliar Si concentrations were not related to their habitat association to moisture (M-value) across all species, even when accounting for phylogenetic relatedness (PGLS: *P* = 0.80, Fig. 4, Table S4), i.e., in contrast to our hypothesis, species associated to drier habitats did not exhibit higher foliar Si concentrations. Nevertheless, two contrasting patterns emerged: the subset of species pairs consisting only of low-accumulating species showed a significant increase of foliar Si with species’ association to drier habitats (PGLS: *P* < 0.001, Fig. 4, Table S4), while the subset of species pairs including at least one high-accumulating showed the opposite relationship, i.e., a significant decrease of foliar Si with species’ association to drier habitats (PGLS: *P* = 0.002).

Foliar Si responses to soil Si availability (RR_Foliar Si_) were also not related to habitat association to moisture regardless of phylogenetic relatedness (Table S4).

## Discussion

### Temperate forbs differ considerably in foliar Si concentrations

Foliar Si concentrations varied substantially across the studied dicotyledonous forbs exhibiting a 70-fold variation from 0.7 to almost 50 mg g^−1^ (equivalent to 0.07–5% Si per dry weight). In our study, environmental factors, which are known to influence plant Si concentrations, can be excluded as a source of this wide variation, since plants were grown on standardized soil under controlled conditions. Differences in foliar Si concentrations should thus reflect genetically based differences among forb species concerning Si uptake and accumulation mechanisms. Maximum foliar Si concentrations in our study were comparable to the highest values observed among 427 non-monocotyledonous angiosperm species in an extensive meta-analysis (Hodson et al. 2005). Even among the monocotyledonous species included in the meta-analysis, especially grasses, which are considered high accumulators, only 3.5% exceeded the values reached in our study species (Hodson et al. 2005). About 20% of the studied forb species can be classified as high-accumulating species (foliar Si > 5 mg g^−1^) and contributed most to the observed interspecific variation in foliar Si concentrations. Previous studies showed similar skewed distributions of Si concentration in dicots, with few high-accumulating and many low-accumulating species (Schaller et al. 2018; Ishizawa et al. 2019; Hodson et al. 2005; Deshmukh et al. 2020). Physiological and ecological benefits of Si in alleviating abiotic and biotic stress, which were initially found in monocots and more recently in dicots (reviewed in Cooke and Leishman 2016; Putra et al. 2020), are assumed to be especially pronounced in high accumulators (Ma 2004). Hence, beneficial effects might vary considerably across forb species. Overall, our results indicate pronounced interspecific variation in foliar Si within dicotyledonous temperate forb species, which may have pervasive yet understudied ecological consequences in grasslands.

### Intraspecific and interspecific variation in foliar Si in response to soil Si availability

Foliar Si concentrations exhibited an intraspecific response to soil Si availability. This intraspecific variation was considerably lower than interspecific variation (coefficient of variation 0.4 and 1.8, respectively). Nevertheless, 19 out of 26 species showed a significant increase (up to fourfold) of foliar Si with higher soil Si availability. The strength of the response differed across species, with species with higher Si accumulation capacity (i.e., high foliar Si at high soil Si) responding stronger to increased soil Si availability than species with lower accumulation capacity. Thus, contrary to our hypothesis, we found no indication that high-accumulating species actively upregulated Si uptake under low soil Si availability to compensate variation of soil Si, and to maintain possible positive effects of high Si concentrations.

Despite the pronounced effect of soil Si availability on foliar Si concentrations, and the difference in species’ responses, species ranking of foliar Si remained consistent across low and high Si soil. Species hierarchies of Si concentrations should thus remain consistent independent of soil Si availability, i.e., species with high Si accumulation capacity should always exhibit high foliar Si concentrations relative to co-occurring species with lower accumulation capacity. Consequently, possible performance-enhancing effects of Si under biotic and abiotic stress should not lead to changes of species performance ranking and competitive hierarchies across habitats with different soil Si availability.

Different responses to soil Si across species should be related to interspecific differences in Si uptake mechanisms. For active uptake mechanisms, variation in the type and number of Si transporters as well as root architecture can contribute to species-specific responses of Si accumulation to soil Si availability (Liang et al. 2006; Ma and Yamaji 2015; Deshmukh and Bélanger 2016). Differences in passive uptake associated with transpiration rates can also influence Si transport through the xylem and its accumulation in leaves; either concurrently to active Si uptake, as shown for high-accumulating species (Liang et al. 2006) or as the only Si uptake process, as in low-accumulating species (Ma et al. 2001; Mitani and Ma 2005). Passive uptake may thus also contribute to interspecific differences of responses (Mitani and Ma 2005; Faisal et al. 2012). In our study species, however, foliar Si concentrations were not positively related with stomatal conductance (unpublished data), indicating that passive processes did not drive the variation. By implication, the observed interspecific variation in the responses of foliar Si concentrations to soil Si availability must be mainly governed by differences in active uptake processes (Liang et al. 2006). While most species showed a significant increase of foliar Si with soil Si availability, seven species (27%) did not respond to soil Si availability. The mechanisms underlying the missing responses of foliar Si concentrations in these species remain open.

The pronounced increase of foliar Si with soil Si availability we found in most species under controlled conditions contrasts with field studies on dicotyledonous species, which showed no effect of soil Si variation on foliar Si (Schaller et al. 2018; Nakamura et al. 2019; Rausch 2021). This suggests that under natural conditions additional environmental factors that modulate foliar Si concentrations, such as water availability, herbivory (Quigley and Anderson 2014; Hartley and DeGabriel 2016) or plant-related factors such as leaf age (Motomura et al. 2002), can override the effect of soil Si on foliar Si concentrations.

### The role of phylogeny for interspecific variation in Si concentrations

At high taxonomic levels, such as class and order, previous studies have shown the importance of phylogeny in driving Si concentrations (Hodson et al. 2005; Katz 2015; Trembath-Reichert et al. 2015; Strömberg et al. 2016). Taxa with high foliar Si concentration in our study, i.e., the Asterales and Boraginaceae, have also been shown to exhibit high Si concentration in Hodson et al. (2005) and Deshmukh et al. (2020) based on different species sets. In this study, the species-level impact of phylogeny on interspecific variation of foliar Si could be specifically addressed due to standardized sampling and growing conditions, the replicated design as well as the selection of phylogenetically diverse species pairs. Our findings emphasize that phylogenetic relatedness has a pronounced effect on species differences in foliar Si concentrations, and consequently on the Si accumulation capacity in forbs. The observation of a phylogenetic signal at species-level within dicots is consistent with the identification of genes that code for active Si transporters and promote high Si concentrations (Ma and Yamaji 2015; Deshmukh and Bélanger 2016; Deshmukh et al. 2020).

Despite the observed prominent phylogenetic signal, closely related species in several of the congeneric and confamiliar species pairs showed pronounced differences in foliar Si concentrations, even suggesting different Si uptake mechanisms. A previous meta-analysis also found large within-genus variation of Si concentrations across grass species, including species assigned to different uptake classes (Strömberg et al. 2016). These results indicate that high Si accumulation (and associated active uptake) has evolved multiple times in forbs and suggest that high Si accumulation may represent an adaptive response (Blomberg and Garland 2002).

### The role of habitat moisture

Si has been shown to alleviate plant drought stress and thus has been suggested to be adaptive in dry habitats (Quigley and Anderson 2014; Katz 2019). If high foliar Si concentrations, or active Si uptake, is an outcome of convergent evolution due to selection by drought, species associated to drier habitats should consistently exhibit higher foliar Si than the species associated to moister habitats across phylogenetically constrained species pairs. Several of the closely related but ecologically divergent species pairs in our study showed substantial differences of foliar Si, but across all species the pronounced interspecific variation in foliar Si concentrations was unrelated to the species’ association to habitat moisture. Thus, our data do not support that in dicotyledonous forbs of temperate grasslands high Si accumulation is generally adaptative under dry conditions. The lack of an overall relationship of foliar Si concentrations with species’ association to habitat moisture also implies that foliar Si concentrations are not a consistent driver of ecological sorting of species across moisture gradients, i.e., forbs with high foliar Si concentrations are associated with a wide range of moisture conditions. Across species, other traits likely override any positive consequences of foliar Si concentrations for drought resistance that have previously been shown within species (Hattori et al. 2008; Cooke and Leishman 2016; Chen et al. 2018), and their consequences for the widely documented filtering of grassland species across moisture gradients (Silvertown et al. 2015).

Interestingly, despite the lack of an overall relationship of foliar Si concentrations to species habitat moisture association, the expected trend (higher foliar Si concentrations in species associated with drier habitats) emerged in the subset of species pairs containing only low accumulating species, while in the subset of pairs that included a high accumulator, higher foliar Si concentrations were associated with moister habitats—opposite to our expectation. This unexpected pattern suggests that the different uptake mechanisms might underlie opposing selection pressures (and lead to opposing ecological sorting) along moisture gradients. Under moist conditions, high Si accumulation (through active uptake) might be advantageous by conferring increased resistance to pest pressure (pathogens or herbivores; Massey et al. 2007; Fauteux et al. 2005). The potential of differing ecological advantages of the different uptake mechanism implied by our results—and contrasting previous findings of general positive effects of Si on plant drought resistance (Hattori et al. 2008; Cooke and Leishman 2016; Chen et al. 2018)—merit further studies.

### Conclusion

Dicotyledonous forb species exhibited a similar range of foliar Si concentrations as grasses. The considerable variation of Si concentrations in forbs likely affects their responses to abiotic and biotic stressors, and may have pervasive, so far unrecognized, ecological consequences for grasslands under land-use and climate change. The lack of an overall relationship of foliar Si concentrations with species’ habitat moisture association, although Si has been assumed to alleviate plant drought stress, suggests that complex interactions between genetically determined Si accumulation as well as abiotic and biotic factors govern ecological effects of Si, and underline the need for further studies. Our study provides a basis for selecting species or taxa for targeted studies on the evolution, and physiological and ecological consequences of Si accumulation.

## Supplementary Information

Below is the link to the electronic supplementary material.

Supplementary file1 (DOCX 55 KB)

## Data Availability

All basic data is deposited in Table S1, S2, S3 and S4 in the supplemental materials. Raw data will also be made available in the DRYAD Digital Depository (https://doi.org/10.5061/dryad.t4b8gtj16), Klotz et al. (2021).
