# Supplementary material for: Variation of foliar silicon concentrations in temperate forbs: effects of soil silicon, phylogeny and habitat
Source: Oecologia. 2021 Jul 14;196(4):977–87. doi: 10.1007/s00442-021-04978-9 (PMC8367921; doi:10.1007/s00442-021-04978-9)
Supplement: Supplementary file 1 — Supplementary file1 (DOCX 55 KB) [file 442_2021_4978_MOESM1_ESM.docx]

**Supplemental Materials**

**Variation of foliar silicon concentrations in temperate forbs: effects of soil silicon, phylogeny and habitat**

**Authors:**

Marius Klotz, Jörg Schaller, Susanne Kurze, Bettina M. J. Engelbrecht

**Contact information of the corresponding author:**Marius Klotz, Department of Plant Ecology, Bayreuth Center of Ecology and Environmental Research (BayCEER), University of Bayreuth, Bayreuth, 95440, Germany**,** Email address: marius.klotz@uni-bayreuth.de**,** Telephone: +49-921-55-2577

**Table S1** Species included in the greenhouse experiment with their name, abbreviation, family, order, Ellenberg indicator value for moisture (M-value; Ellenberg et al. 2001), and the number of replicates for the high and low Si treatment (N Si+, N Si-).

| **Species** | **Family** | **Order** | **N (Si +)** | **N (Si -)** | **M-value** | **Abbreviation** |
| --- | --- | --- | --- | --- | --- | --- |
| *Alchemilla xanthochlora* Rothm.*** | Rosaceae | Rosales | 5 | 5 | 7 | Alxa |
| *Alyssum alyssoides*(L.) L. | Brassicaceae | Brassicales | 5 | 5 | 3 | Alal |
| *Anchusa officinalis* L. | Boraginaceae | Boraginales | 5 | 5 | 3 | Anof |
| *Artemisia campestris* L. | Asteraceae | Asterales | 5 |  | 2 | Arca |
| *Artemisia vulgaris* L. | Asteraceae | Asterales | 5 | 5 | 6 | Arvu |
| *Barbarea vulgaris* R. Br., s.l. | Brassicaceae | Brassicales | 5 |  | 6 | Bavu |
| *Campanula patula* L. | Campanulaceae | Asterales | 5 | 5 | 5 | Capa |
| *Campanula rapunculus* L. | Campanulaceae | Asterales | 5 | 5 | 3 | Cara |
| *Centaurea pseudophrygia* C. A. Mey.*** | Asteraceae | Asterales | 5 | 5 | 5 | Ceps |
| *Centaurea scabiosa* L. | Asteraceae | Asterales | 5 | 5 | 3 | Cesc |
| *Dianthus armeria* L. | Caryophyllaceae | Caryophyllales | 5 | 5 | 5 | Diar |
| *Dianthus deltoides* L. | Caryophyllaceae | Caryophyllales | 5 | 5 | 3 | Dide |
| *Epilobium angustifolium* L.*** | Onagraceae | Myrtales | 5 |  | 5 | Epan |
| *Epilobium hirsutum* L. | Onagraceae | Myrtales | 5 | 5 | 8 | Ephi |
| *Galium album* Mill. | Rubiaceae | Gentianales | 5 | 5 | 5 | Gaal |
| *Hypericum maculatum* Crantz, s. str. | Hypericaceae | Malpighiales | 5 | 5 | 6 | Hyma |
| *Hypericum perforatum* L. | Hypericaceae | Malpighiales | 5 | 5 | 4 | Hype |
| *Knautia arvensis* (L.) Coult., s. str. | Caprifoliaceae | Dipsacales | 5 |  | 4 | Knar |
| *Lathyrus pratensis* L. | Fabaceae | Fabales | 5 |  | 6 | Lapr |
| *Lathyrus tuberosus* L. | Fabaceae | Fabales | 5 |  | 4 | Latu |
| *Myosotis arvensis* (L.) Hill | Boraginaceae | Boraginales | 5 | 4 | 5 | Myar |
| *Pimpinella major* (L.) Huds.*** | Apiaceae | Apiales | 3 |  | 5 | Pima |
| *Pimpinella saxifraga* L. | Apiaceae | Apiales | 4 |  | 3 | Pisa |
| *Potentilla recta* L. | Rosaceae | Rosales | 5 | 5 | 3 | Pore |
| *Primula elatior* (L.) Hill | Primulaceae | Ericales | 5 |  | 6 | Prel |
| *Primula veris* L. | Primulaceae | Ericales | 5 |  | 4 | Prve |
| *Prunella grandiflora* (L.) Scholler | Lamiaceae | Lamiales | 5 | 5 | 3 | Prgr |
| *Prunella vulgaris* L. | Lamiaceae | Lamiales | 5 | 5 | 5 | Prvu |
| *Ranunculus acris* L. | Ranunculaceae | Ranunculales | 5 |  | 6 | Raac |
| *Rumex thyrsiflorus* Fingerh. | Polygonaceae | Caryophyllales | 5 | 5 | 3 | Ruth |
| *Silene flos-cuculi* (L.) Clairv. | Caryophyllaceae | Caryophyllales | 5 | 5 | 7 | Sifl |
| *Silene viscaria* (L.) L. | Caryophyllaceae | Caryophyllales | 5 | 5 | 3 | Sivi |
| *Trifolium pratense* L. | Fabaceae | Fabales | 5 | 5 | 5 | Trpr |
| *Verbascum lychnitis* L.*** | Scrophulariaceae | Lamiales | 4 | 5 | 3 | Vely |
| *Verbascum nigrum* L. | Scrophulariaceae | Lamiales | 5 | 5 | 5 | Veni |
| *Veronica longifolia* L., s. l. | Plantaginaceae | Lamiales | 5 | 5 | 8 | Velo |
| *Veronica teucrium* L.*** | Plantaginaceae | Lamiales | 5 | 5 | 3 | Vete |
| ***** For phylogenetic analysis six study species that were missing in the phylogeny by Zanne et al. (2014) were replaced by congeneric species. If available, we consulted genus-specific phylogenies from the literature (for *Pimpinella*: Wang et al. 2014, for *Epilobium*: Baum et al. 1994). | | | | | | |

**Table S2** Foliar Si concentrations under low soil Si availability (non-manipulated soil) and under high soil Si availability (Si-enriched soil; mean ± standard deviation) and species’ foliar Si response to soil Si availability (RR_Foliar Si_) in 26 temperate dicots. F-value and *P*-value of the one-way ANOVA testing the effect of soil Si (based on log_10_ transformed foliar Si concentrations) within each species are given (for n see Table S1). Degrees of freedom was 1 for the numerator, and 8 for the denominator (except 7 in species *Myosotis arvensis* and *Verbascum lychnitis*). Significant *P*-values are marked in bold.

| **Species** | **Foliar Si concentration (mg g^-1^)** | | **RR_Foliar Si_** |  | **Effect of soil Si** | |
| --- | --- | --- | --- | --- | --- | --- |
|  | **low Si** | **high Si** |  |  | **F-value** | ***P*-value** |
| *Alchemilla xanthochlora* | 2.60 ± 0.36 | 10.77 ± 1.03 | 0.618 |  | 384.806 | **< 0.001** |
| *Alyssum alyssoides* | 1.28 ± 0.55 | 1.34 ± 0.34 | 0.018 |  | 0.162 | 0.698 |
| *Anchusa officinalis* | 5.79 ± 0.71 | 19.03 ± 3.13 | 0.517 |  | 181.385 | **<0.001** |
| *Artemisia campestris* |  | 4.17 ± 0.60 |  |  |  |  |
| *Artemisia vulgaris* | 3.85 ± 0.30 | 12.26 ± 2.73 | 0.503 |  | 97.266 | **< 0.001** |
| *Barbarea vulgaris* |  | 0.67 ± 0.29 |  |  |  |  |
| *Campanula patula* | 3.09 ± 0.56 | 6.19 ± 1.57 | 0.302 |  | 20.506 | **0.002** |
| *Campanula rapunculus* | 1.01 ± 0.19 | 1.59 ± 0.40 | 0.197 |  | 10.156 | **0.013** |
| *Centaurea pseudophrygia* | 2.71 ± 0.52 | 9.50 ± 1.15 | 0.545 |  | 151.134 | **< 0.001** |
| *Centaurea scabiosa* | 1.45 ± 0.34 | 3.46 ± 0.91 | 0.377 |  | 27.253 | **< 0.001** |
| *Dianthus armeria* | 0.75 ± 0.45 | 1.01 ± 0.18 | 0.132 |  | 3.180 | 0.112 |
| *Dianthus deltoides* | 1.10 ± 0.37 | 2.41 ± 0.96 | 0.342 |  | 10.144 | **0.013** |
| *Epilobium angustifolium* |  | 1.06 ± 0.20 |  |  |  |  |
| *Epilobium hirsutum* | 0.56 ± 0.28 | 0.80 ± 0.42 | 0.150 |  | 1.569 | 0.246 |
| *Galium album* | 6.09 ± 0.79 | 25.35 ± 6.32 | 0.619 |  | 116.111 | **< 0.001** |
| *Hypericum maculatum* | 0.35 ± 0.13 | 0.74 ± 0.14 | 0.324 |  | 19.461 | **0.002** |
| *Hypericum perforatum* | 0.58 ± 0.09 | 0.69 ± 0.07 | 0.074 |  | 5.660 | **0.045** |
| *Knautia arvensis* |  | 0.84 ± 0.34 |  |  |  |  |
| *Lathyrus pratensis* |  | 1.34 ± 0.16 |  |  |  |  |
| *Lathyrus tuberosus* |  | 2.10 ± 0.60 |  |  |  |  |
| *Silene flos-cuculi* | 0.50 ± 0.07 | 0.73 ± 0.09 | 0.166 |  |  |  |
| *Myosotis arvensis* | 20.64 ± 4.63 | 47.92 ± 6.62 | 0.366 |  | 55.062 | **< 0.001** |
| *Pimpinella major* |  | 0.99 ± 0.23 |  |  |  |  |
| *Pimpinella saxifraga* |  | 1.06 ± 0.38 |  |  |  |  |
| *Potentilla recta* | 0.60 ± 0.05 | 0.81 ± 0.14 | 0.131 |  | 11.366 | **0.010** |
| *Primula elatior* |  | 1.45 ± 0.23 |  |  |  |  |
| *Primula veris* |  | 2.36 ± 1.19 |  |  |  |  |
| *Prunella grandiflora* | 0.98 ± 0.62 | 1.24 ± 0.32 | 0.102 |  | 1.582 | 0.244 |
| *Prunella vulgaris* | 0.39 ± 0.01 | 0.75 ± 0.11 | 0.279 |  | 78.719 | **< 0.001** |
| *Ranunculus acris* |  | 11.09 ± 3.34 |  |  |  |  |
| *Rumex thyrsiflorus* | 0.48 ± 0.09 | 0.98 ± 0.15 | 0.308 |  | 41.204 | **< 0.001** |
| *Silene viscaria* | 0.96 ± 0.30 | 1.35 ± 0.48 | 0.149 |  | 2.358 | 0.163 |
| *Trifolium pratense* | 0.49 ± 0.12 | 0.86 ± 0.14 | 0.246 |  | 17.072 | **0.003** |
| *Veronica longifolia* | 0.59 ± 0.22 | 0.74 ± 0.19 | 0.099 |  | 1.867 | 0.209 |
| *Verbascum lychnitis* | 0.67 ± 0.66 | 0.98 ± 0.80 | 0.165 |  | 0.926 | 0.368 |
| *Verbascum nigrum* | 0.41 ± 0.12 | 0.73 ± 0.10 | 0.248 |  | 19.919 | **0.002** |
| *Veronica teucrium* | 1.49 ± 0.35 | 2.68 ± 0.71 | 0.256 |  | 9.974 | **0.013** |

**Table S3** F-value and *P*-value of the one-way ANOVA testing the differences in foliar Si concentration (based on log_10_ transformed foliar Si concentrations) within each species pair (for n see Table S1). Degrees of freedom was 1 for the numerator, and 8 for the denominator (except 7 in Velc *vs.* Veni and 5 in Pima *vs.* Pisa). Significant *P*-values are marked in bold. See Table S1 for species abbreviations.

| **Species pair** | **F-value** | ***P*-value** |
| --- | --- | --- |
| Prgr *vs .*Prvu | 12.976 | **0.007** |
| Vely *vs.* Veni | 0.124 | 0.735 |
| Velo *vs.* Vete | 48.600 | **< 0.001** |
| Anof *vs.* Myar | 97.265 | **< 0.001** |
| Arca *vs.* Arvu | 68.199 | **< 0.001** |
| Ceps *vs.* Cesc | 50.542 | **< 0.001** |
| Capa *vs.* Cara | 64.197 | **< 0.001** |
| Pima *vs.* Pisa | 0.040 | 0.849 |
| Prel *vs.* Prve | 3.318 | 0.106 |
| Sifl *vs.* Sivi | 11.168 | **0.010** |
| Diar *vs.* Dide | 15.652 | **0.004** |
| Lapr *vs.* Latu | 6.892 | **0.030** |
| Alxa *vs.* Pore | 856.163 | **< 0.001** |
| Hyma *vs.* Hype | 0.385 | 0.552 |
| Alal *vs.* Bavu | 13.320 | **0.006** |
| Epan *vs.* Ephi | 2.964 | 0.123 |

**Table S4** Summary of regression analyses of (a) foliar Si (high Si treatment) or (b) response to soil Si availability (RR_Foliar Si_) on species’ habitat association to moisture (M-value, Ellenberg et al. 2001). Results are given for ordinary least square regression (OLS), and for phylogenetic generalized least square regression (PGLS) with a λ-modified variance-covariance matrix. Given are the regression slope (β), *P*-value, AIC and Pagels’ λ. The model with the highest fit (lowest AIC value) is indicated in bold. We dismissed Brownian motion PGLSs despite a significant slope because the model comparison based on AIC-values consistently indicated the lowest fit among the calculated models. We additionally present the respective results for separate analyses of foliar Si on species’ habitat association to moisture for (c) those species pairs including only low-accumulating species and (d) species pairs containing high-accumulating species.

| 1. **Effect of habitat association to moisture on foliar Si (all species pairs)** | | | | | |
| --- | --- | --- | --- | --- | --- |
|  |  | **β** | ***P*** | **AIC** | **λ** |
| **OLS** |  | -0.033 | 0.544 | 55.731 |  |
| **PGLS** | *λ* | **-0.009** | **0.798** | **48.526** | **0.788** |
|  |  |  |  |  |  |
| 1. **Effect of habitat association to moisture on RR_Si_** | | | | | |
|  |  | **β** | ***P*** | **AIC** | **λ** |
| **OLS** |  | **0.012** | **0.593** | **-2.173** |  |
| **PGLS** | *λ* | 0.014 | 0.505 | -0.724 | 0.363 |
|  |  |  |  |  |  |
| 1. **Effect of habitat association to moisture on foliar Si (species pairs including only low-accumulating species)** | | | | | |
|  |  | **β** | ***P*** | **AIC** | **λ** |
| **OLS** |  | -0.065 | 0.007** | -3.017 |  |
| **PGLS** | *λ* | **-0.084** | **< 0.001***** | **-5.455** | **0.811** |
|  |  |  |  |  |  |
| 1. **Effect of habitat association to moisture on foliar Si (species pairs including high-accumulating species)** | | | | | |
|  |  | **β** | ***P*** | **AIC** | **λ** |
| **OLS** |  | 0.167 | 0.124 | 22.107 |  |
| **PGLS** | *λ* | **0.172** | **0.002**** | **18.243** | **0.922** |

**References**

Baum DA, Sytsma KJ, Hoch PC (1994) A phylogenetic analysis of *Epilobium* (Onagraceae) based on nuclear ribosomal DNA Sequences. Syst Bot 19:363. <https://doi.org/10.2307/2419763>

Ellenberg H, Weber HE, Düll R, Wirth V, Werner W (2001) Zeigerwerte von Pflanzen in Mitteleuropa, 3rd edn. Verlag Erich Goltze GmbH & Co KG, Göttingen.

Wang Z-X, Downie SR, Tan J-B, Liao C-Y, Yu Y, He X-J (2014) Molecular phylogenetics of *Pimpinella* and allied genera (Apiaceae), with emphasis on Chinese native species, inferred from nrDNA ITS and cpDNA intron sequence data. Nord J Bot 32:642–657. <https://doi.org/10.1111/j.1756-1051.2013.00343.x>

Zanne AE, Tank DC, Cornwell WK, Eastman JM, Smith SA, FitzJohn RG, McGlinn DJ, O‘Meara BC, Moles AT, Reich PB, Royer DL, Soltis DE, Stevens PF, Westoby M, Wright IJ, Aarssen L, Bertin RI, Calaminus A, Govaerts R, Hemmings F, Leishman MR, Oleksyn J, Soltis PS, Swenson NG, Warman L, Beaulieu JM (2014) Three keys to the radiation of angiosperms into freezing environments. Nature 506:89–92. <https://doi.org/10.1038/nature12872>
